# AVC: Selecting discriminative features on basis of AUC by maximizing variable complementarity

**DOI:** 10.1186/s12859-017-1468-4

**Published:** 2017-03-14

**Authors:** Lei Sun, Jun Wang, Jinmao Wei

**Affiliations:** Institute of Big Data, College of Computer and Control Engineering, Nankai University, 38 Tongyan Road, Tianjin, 300350 China

**Keywords:** Feature selection, ROC curve, AUC, Feature complementarity

## Abstract

**Background:**

The Receiver Operator Characteristic (ROC) curve is well-known in evaluating classification performance in biomedical field. Owing to its superiority in dealing with imbalanced and cost-sensitive data, the ROC curve has been exploited as a popular metric to evaluate and find out disease-related genes (features). The existing ROC-based feature selection approaches are simple and effective in evaluating individual features. However, these approaches may fail to find real target feature subset due to their lack of effective means to reduce the redundancy between features, which is essential in machine learning.

**Results:**

In this paper, we propose to assess feature complementarity by a trick of measuring the distances between the misclassified instances and their nearest misses on the dimensions of pairwise features. If a misclassified instance and its nearest miss on one feature dimension are far apart on another feature dimension, the two features are regarded as complementary to each other. Subsequently, we propose a novel filter feature selection approach on the basis of the ROC analysis. The new approach employs an efficient heuristic search strategy to select optimal features with highest complementarities. The experimental results on a broad range of microarray data sets validate that the classifiers built on the feature subset selected by our approach can get the minimal balanced error rate with a small amount of significant features.

**Conclusions:**

Compared with other ROC-based feature selection approaches, our new approach can select fewer features and effectively improve the classification performance.

## Background

Microarray gene expression data has been analyzed in a wide variety of problems in bioinformatics fields. An important application is to develop a classifier to discriminate instances of different classes [1]. Some classification approaches in machine learning have been applied on the microarray data sets, such as Support Vector Machine (SVM), k-Nearest Neighbor (KNN), Naive Bayes, etc. The published microarray data sets, such as colon tumor [2], GLI-85/GSE4412 [3], and breast cancer [4], usually have high dimensionalities and small sample sizes because of the significant cost and effort required to collect and genotype specimens. For microarray data sets with ten thousands of genes but only tens of observations (instances), reducing the high-dimensional gene space is an important issue in terms of classification. Not all the genes make significant contributions to recognizing the target diseases, and only a few of genes with multiple genomic mutations determine biological or clinical properties [5]. Gene selection can interpret the original characteristics of genes and improve the performance of classification by removing the irrelevant and redundant genes [6]. Gene selection is equivalent to feature selection in pattern recognition and machine learning. Many feature selection approaches have been used to select genes. Traditional gene selection approaches rank genes based on some classic criteria, including t-test [7], non-parametric statics [8], *P*-value [9], information gain [10], etc. They can find the excellent genes and select the top ranked ones for discriminating the target diseases. Recently, many effective approaches utilizing the filter evaluation framework have been studied by researchers [11–15].

The ROC curve which is strongly related with non-parametric hypothesis testing has shown special attractiveness. As a non-parametric measure, ROC curve has exhibited favorable evaluation characteristics on the imbalanced and cost-sensitive data classification problems [16]. This superiority is obtained mainly because ROC curve compares classifiers’ performance through the entire range of class distributions and error costs. The ROC curve and AUC (area under the ROC curve) have been widely used to determine the classification accuracy in supervised learning [17]. Through analyzing a two-dimensional graph, it is hard to compare two ROC curves directly. AUC, which is denoted as a quantitative measurement, provides a good summary for examining the ROC curves [18]. As a scalar measure, AUC has been widely exploited to evaluate the relevance between features and target class in feature selection approaches, especially for the microarray data sets [16, 19–21].

Since ROC curve and AUC are effective in selecting discriminative features that make less recognition errors, dozens of feature selection approaches are proposed based on the two metrics. The Feature Assessment by Sliding Thresholds (FAST) approach [16] and the statistical gene ranking approach [20] use the technique of ROC analysis to measure the relevance of features with the target class. They evaluate features by calculating the AUCs of the single feature classifiers and then sort them in a descending order according to their AUC values. The top-ranked features are selected into the feature subset. However, a significant flaw is that the selected features may highly correlate with each other, which are sometimes too redundant to be fed into a classifier. The AUC and Rank Correlation coefficient Optimization (ARCO) approach [19] and the Feature selection based-on ROC-curves (FROC) approach [21] are both ROC-based feature selection approaches, which consider the redundancy analysis that cannot be solved in FAST. In ARCO, the redundancy between features is measured by the Spearman’s Rank Correlation Coefficient (RCC). Features with maximum AUC and minimum RCC are selected into the feature subset. However, RCC determines all instances’ ranks on two features without differentiating whether or not the instances are misclassified by the single feature classifiers. This leads to an inevitable problem, that is, redundant features may also have small RCCs due to the instances which can be correctly classified by the single feature classifiers. In FROC, features are ranked according to the area between the ROC curve and the diagonal line (ARD) which is equal to *AUC*−0.5, and then the redundant features are eliminated using the Markov blanket analysis. Note that the redundancy between a pair of features is measured and reduced in terms of the area between the ROC curves (ABR) by FROC. For each feature in the candidate subset, FROC computes its ABR with other features, and the feature with minimal ABR will be removed. This approach can find pairwise redundant features from the candidate subset, yet which one should be removed still remains a difficult problem.

The aforementioned approaches mainly focus on alleviating the redundant information of features, but ignore the global classification performance of the combination of the irredundant features. The ROC curve of one feature may go above or under the curve of another feature, which may convey that this one is more or less discriminative than the other one. When the two curves cross, two features show to be complementary to each other in classification. When analyzing two features as given one feature as selected, we are only interested in whether another one is complementary in classifying the instances that the selected one cannot classify. In this case, the ABR measure in FROC turns to be inapplicable. This leads to the notion of feature complementarity, which is in some sense closely related to feature redundancy. From the classification perspective, complementarity evaluates whether a combination of features can return more joint information about the target class rather than the information carried by each feature individually [22]. Intuitively, instead of examining the relevance between features for determining whether or not one is redundant with another, feature complementary is more direct and applicable in ascertaining the global classification abilities of the selected features. It is a promising way to improve the recognition performance of the ROC-based approaches by evaluating feature complementarity for classification. In view of the above analysis, we propose a new feature selection approach based on the ROC analysis for feature complementarity in this paper.

The proposed approach, named feature selection with AUC-based Variable Complementarity (AVC), uses the technique of ROC analysis to assess the relevance of features with the target class. Moreover, it exploits the information of the instances misclassified by the single feature classifiers based on the ROC curve to analyze the complementarity of features. Apparently, when taking an individual feature as the observation dimension, more or less instances will be misclassified. Thus, we lay the emphasis on the common misclassified instances for two features when evaluating their complementarity for classification. One nearest neighbor from different class (nearest miss) for each common misclassified instance is found out with respect to each feature. Then, two Manhattan distances for each common misclassified instance to its two nearest misses are compared, and the larger one is adopted to calculate the complementarity of the features. It should be pointed out that such technology of analyzing the nearest neighbors is also adopted by some state-of-the-art feature selection methods, such as ReliefF [23], LLBFS [11], nnFRFS [24], etc. Intuitively, we average these Manhattan distances for all the common misclassified instances and exploit them as two features’ complementarity. The instances misclassified by both features are focused on to lay stress on their influences on the accuracies of the classifiers. And the impacts of the instances that can be classified correctly by both features are reduced, because these instances provide little valuable information for recognizing the target class. In addition, we use the greedy sequential forward search approach to find the optimal feature subset, in which classes are maximally separated from each other. This issue is critical for enhancing the global discriminative performance of the selected feature subset. We compare our approach with four state-of-the-art feature selection approaches, that is, three popular approaches based on the ROC curve, FAST, ARCO and FROC, and one well-known approach ReliefF. The experimental results on a broad range of the microarray data sets show that our approach can effectively select small feature subsets, and the performance of the classifiers built on these subsets is obviously improved.

## Methods

A complicated problem in the ROC-based feature selection methods mentioned above is that the feature subsets selected by the existing methods cannot promise the global optimal performance for recognizing the target classes. To overcome this problem, we present a new feature selection method based on the AUC and variable (feature) complementarity analysis, which is called as feature selection with AUC-based Variable Complementarity (AVC). AVC combines the feature relevance and feature complementarity by making the best use of the non-parametric property of AUC. In this section, we describe AVC on the binary-class problem first, and then extend it to the multi-class problem.

Before pinning down the method, some notions are lists as follows:

- **X**: the set of the instances, containing *n* instances $\{\mathbf {x}_{i}\}_{i=1}^{n}$ characterized by *m* features $\mathbf {F}=\{\mathbf {f}_{j}\}_{j=1}^{m}$, and *x*_*ij*_ is the instance **x**_*i*_’s observation value on the feature **f**_*j*_.
- **C**: the set of the classes, including *q* classes **C**={**c**_1_ …,**c**_*q*_}∈*I* *R* ^n×q^.
- *n*_0_,*n*_1_: the number of the positive instances and the negative instances in the data set. Note that *n*=*n*_0_+*n*_1_.

### ROC curve

ROC curve was first used in signal detection theory to represent the tradeoff between the hit rates and false alarm rates. It has been extensively studied and applied in medical diagnosis and evaluation of machine learning algorithms [18]. ROC curves are two-dimensional graphs in which true positive rate (TPR) is plotted on the Y-axis and false positive rate (FPR) is plotted on the X-axis. The good performance of a classifier is reflected by an ROC curve which lies in the upper left triangle of the square. AUC provides a value description for the performance of the ROC curve. AUC is a portion of the area of the unit square, so its value will always between 0 and 1, and usually larger than 0.5 [25]. Due to its several nice properties, AUC has been used in feature selection for microarray analysis. Firstly, AUC is insensitive to the costs unknown problem, because it focuses on the comparison of the distributions of two classes. Secondly, AUC can be used to reflect how well the feature differentiates between the distributions of two classes. Thirdly, AUC is a non-parametric measure index, which is obtained by counting the TPR and FPR of the given samples. So it is appropriate to class imbalanced and costs unknown problems especially in bioinformatics. Besides, the AUC measure of performance is closely related to the Gini coefficient [26], which is most commonly defined as twice the area between the ROC curve and the diagonal (*Gini*+1=2×*AUC*).

Consider a binary classification problem with *n* instances and *m* features. To generate the ROC curve of a classifier, the classifier gives every instance an estimated probability $\hat {p}$, that represents the degree to which an instance is a member of a class. There is a threshold *t* and the instances whose $\hat {p}$ are larger than *t* are predicted as positive class and others are predicted as negative class. For a fixed threshold *t*, there is a point (FPR, TPR) in ROC space. If we vary *t* from 0 to 1, and calculate TPR and FPR at each *t*, we can get the ROC curve of the classifier. To computing AUC, a direct method is to measure the area by applying a rectangle or trapezoid area on each point. But this is too complex and costly. Hand, et al. [26] has proposed a simple method to compute the AUC. In this method, the instances are sorted in increasing order according to their $\hat {p}$. And the AUC is calculated according to the Eq. (1):

1 $$\begin{array}{@{}rcl@{}} AUC=\frac{\sum\limits_{i=1}^{n_{0}}(r_{i}-i)}{n_{0}\times n_{1}}=\frac{\sum\limits_{i=1}^{n_{0}}r_{i}-\frac{n_{0}\times(n_{0}+1)}{2}} {n_{0}\times n_{1}}, \end{array} $$

where *r*_*i*_ is the rank of the *i*th positive instance in the ranked list, and *n*_0_ and *n*_1_ are the numbers of the positive and negative instances. This method shows that AUC is equivalent to the probability that a randomly chosen positive instance will have a higher estimated probability of belonging to the positive class than a randomly chosen negative instance.

In the cases of multi-class classification problems, there have been many extensions to the multi-class AUC such as the average weighted AUC [27] and the volume under the ROC surface [28]. A simple generalisation formulation of AUC for multi-class classification problems was proposed in [26]. It has been widely used to evaluate the performance of classifiers [29]. MAUC directly divides a multi-class problem with *q* classes into $\frac {q(q-1)}{2}$ binary-class sub-problems. AUC of a binary-class sub-problem with the *i*th and *j*th class are represented by *AUC*_*ij*_ and *AUC*_*ji*_. They are calculated by Eq. (1) with the *i*th and *j*th class seen as positive class respectively. MAUC is calculated according to the Eq. (2):

2 $$\begin{array}{@{}rcl@{}} MAUC=\frac{1}{q(q-1)}\sum\limits_{i<j}\left[AUC_{ij}+AUC_{ji}\right]. \end{array} $$

In the feature selection problem, when a method uses AUC as the metric to evaluate the relevance between a feature and target class, the instances’ values for this feature are viewed as the output of a classifier which is equivalent to $\hat {p}$. If a feature is irrelevant to the target class, its AUC is close to 0.5, and if a feature is highly relevant to the target class, its AUC is closer to 1. We use *AUC*(**f**_*i*_) for binary-class problem and *MAUC*(**f**_*i*_) for multi-class problem to represent the AUC of feature **f**_*i*_ in this paper.

### Binary-class problem

In feature selection, a single feature’s predictive power can be ascertained according to this feature’s classification performance taken individually as a classifier [30]. The single feature classifier built by feature **f**_*j*_ can choose a proper threshold *θ*. If *x*_*ij*_≥*θ*,**x**_*i*_ is classified into the positive class. And if *x*_*ij*_<*θ*,**x**_*i*_ is classified into the negative class. This critical parameter *θ* can be determined in terms of some metrics, such as AUC, classification accuracy, etc. In this paper, AUC is used to measure features’ predictive power which is superior in the evaluation of imbalanced and cost-sensitive data.

**Fig. 1:**
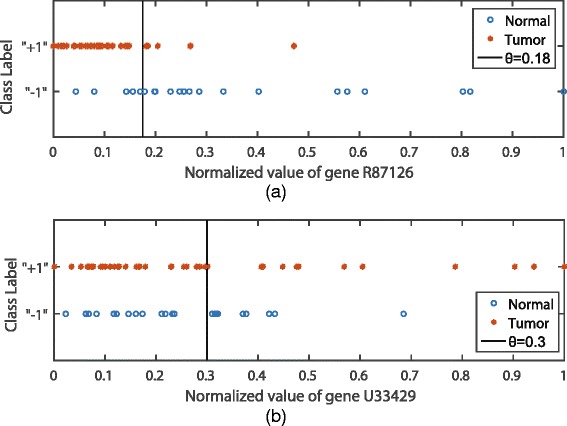
One-dimensional instances distribution. Different distribution of instances on two features. The *vertical lines* represent the threshold *θ* of two single feature classifiers of two features. (**a**) shows the distribution of instances on gene R87126 and (**b**) shows the distribution of instances on gene U33429

Similar with ARCO, we also employ the AUC of a single feature as the relevance metric. Instances are ranked according to their observation values on feature **f**_*i*_. And then, *AUC*(**f**_*i*_) is calculated with Eq. (1). Figure 1 shows an example of the microarray data set Colon [2] for further illustrating the characteristic of AUC. We can observe from Fig. 1 (a) that, when *θ*=0.18, a majority of instances can be correctly divided into two classes on the gene R87126. In Fig. 1 (b), only about half of instances can be correctly divided into two classes on the gene U33429. Even though when *θ*=0.3, the maximal classification accuracy obtained by the gene U33429 as a single feature classifier is equal to 0.6. Correspondingly, we can calculate the AUCs of two features by Eq. (1) as *AUC*(**f**_*i*_)=0.884 and *AUC*(**f**_*i*_) =0.5. Considering the existing feature selection methods based-on ROC curve, the larger the *AUC*(**f**_*i*_) is, the more relevant feature **f**_*i*_ is with the target class. Thus, we can assume that gene R87126 is more relevant than gene U33429.

Using AUC as the criterion to measure the relevance of features and target class can find the most significant features to discriminate the given classes, but these features are sometimes too redundant to be inputted to a classifier. Different from the existing ROC-based feature selection methods which reduce feature redundancy, our approach AVC analyzes features’ complementarity, which denotes the joint classification information provided by features. It is more or less than the sum of the information taken by features individually. Our aim is to find out the most complementary features that jointly provide maximal classification information [22].

**Fig. 2:**
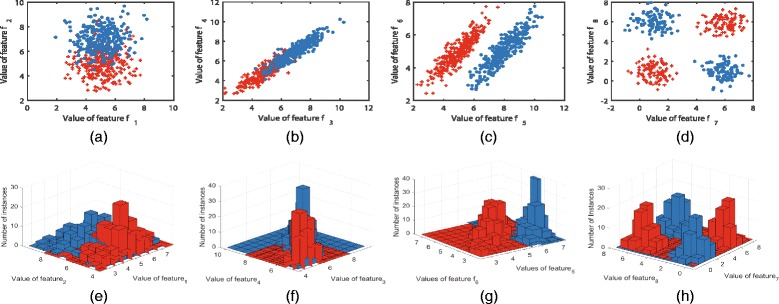
Illustration of feature complementarity. 2-dimensional instances’ distributions on different combination of features: (**a**) to (**d**) describe the class distributions when the instances are projected to different pairwise features, and (**e**) to (**h**) are the corresponding histograms

In order to show the importance of feature complementarity, we take Fig. 2 as an example. In Fig. 2, a group of artificial data sets containing 200 random instances characterized by different pairwise features are constructed. Figure 2 (a) to (d) show the class distributions in different two-dimensional feature space. The histograms of the instances projected on the subspace constructed by the corresponding two features are demonstrated in Fig. 2 (e) to (h), respectively. Note that both classes have the same number of instances and submit to the Gaussian distributions with equal covariance. It can be observed that when projecting the instances to different pairwise features, the class distributions are rather different. In Fig. 2 (a), the distributions of the two classes overlap between each other. It means that a majority of the instances belonging to the two classes cannot be correctly recognized in the subspace constructed by feature **f**_1_ and **f**_2_. In Fig. 2 (b), the class conditional distributions have a high covariance in the direction of the line of the two class centers. We can see that classes also cannot be separated in the subspace of feature **f**_3_ and **f**_4_. Compared with Fig. 2 (a) and (b), (c) shows a special case, that is, one feature has completely overlapping class distributions. It means that neither feature **f**_5_ nor feature **f**_6_ can scatter two classes individually. Yet all the instances can be correctly classified in the subspace collaboratively constructed by **f**_5_ and **f**_6_. Another special case is given in Fig. 2 (d), in which two classes overlap perfectly no matter projected on feature **f**_7_ or feature **f**_8_. Similar with the case in Fig. 2 (c), they can be separated perfectly in the subspace of the two features. Thus, we can draw the conclusion from the subfigures (c) and (d) that, two individually inferior features can be superior when combined together. The histograms in Fig. 2 (e) to (h) also exhibit this property as in Fig. 2 (a) to (d). Therefore, even if some individual features may have bad separability capabilities, their combinational feature subset may probably provide good class separability performance. Just on the basis of this important characteristics of the features, our new approach AVC pays emphasis on the complementarity between features in pair, which is expected to effectively improve the classification performance of the selected feature subset.

It is critical to analyze the data distributions on pairwise features to evaluate the complementarity between them. As aforementioned, a feature’s AUC indicates the distribution of the positive class and negative class on this feature dimension. If all the positive class instances rank higher than the negative class instances, AUC will be equal to 1, which means that all the instances can be correctly classified into two classes. If a feature’s AUC is smaller than 1, it implies that more or less instances will be misclassified by this single feature classifier. For a data set with *n* instances, there exist *n*_0_×*n*_1_ instance pairs, in which a positive instance and a negative instance are simultaneously included. The special pairs in which the positive instances are ranked higher than the negative ones are drawn attentions from AUC. AUC actually denotes the ratio of these special pairs out of all the instance pairs. In the pair of instances that positive class instance ranked lower than the negative class instance, there must be a misclassified instance. We focus on the distribution of these misclassified cases under the different combination of features to find out the features which have the maximal complementarity of classification capability such as the features in Fig. 2 (c) and (d). The basic idea is, if the instances from different classes that are close to each other on one feature dimension are far apart on another feature dimension, the two features are regarded as complementary to each other. In order to find out such features, we introduce a new metric to evaluate the complementarity between two features. This metric is based on the similarity of instances inspired by the state-of-the-art feature selection method ReliefF [23], which adopts the nearest neighbor rule to evaluate features. We use the nearest neighbor rule on the set of the misclassified instances according to the single feature classifiers to analyze the complementarity between two features. Specifically, the average Manhattan distance between the misclassified instances and their nearest neighbors from the other class (nearest miss) are exploited to represent the complementarity between two features.

We use the matrix $\mathcal {H}$ to represent the complementarity of the feature classification capability as follows:

3 $$\begin{array}{@{}rcl@{}} \mathcal{H}\triangleq\left(\begin{array}{ccccc} 0 & h_{12} & h_{13} & \ldots & h_{1m} \\ 0 & 0 & h_{23} & \ldots & h_{2m} \\ \vdots & \vdots & \vdots & \ddots & \vdots\\ 0 & 0 & 0 & \ldots & h_{(m-1)m} \\ 0 & 0 & 0 & \ldots & 0 \\ \end{array} \right), \end{array} $$

where *h*_*ij*_ is the complementarity between feature **f**_*i*_ and **f**_*j*_, defined as:

4 $$\begin{array}{@{}rcl@{}} \begin{aligned} & h_{ij}=\frac{\sum\limits_{k=1}^{|\mathbf{S}|}d_{k}\cdot MD\left(\mathbf{x}_{k},\mathbf{I}_{ik},\mathbf{I}_{jk}\right)} {|\mathbf{S}|},\;\\ & d_{k}=\left\{ \begin{array}{ll} \!0,\ \mathbf{I}_{ik}=\mathbf{I}_{jk}\\ \!1,\ \mathbf{I}_{ik}\neq\mathbf{I}_{jk} \end{array},\right. \\ & MD\left(\mathbf{x}_{k},\mathbf{I}_{ik},\mathbf{I}_{jk}\right)=\max\left(dis(\mathbf{x}_{k},\mathbf{I}_{ik}), dis(\mathbf{x}_{k},\mathbf{I}_{jk})\right), \end{aligned} \end{array} $$

where **S** is the intersection of instances misclassified by both feature **f**_*i*_ and **f**_*j*_, and **x**_*k*_ is an instance in **S**. **I**_*ik*_ and **I**_*jk*_ are **x**_*k*_’s nearest misses respectively obtained from the angle of features **f**_*i*_ and **f**_*j*_, and *dis*(·,·) is the Manhattan distance between the two involved variables.

**Fig. 3:**
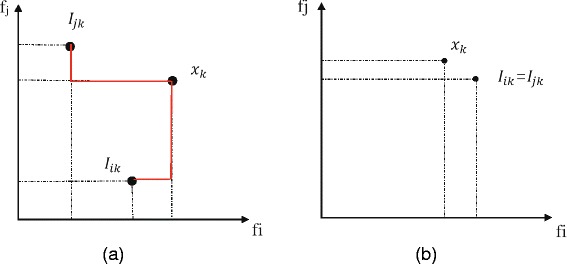
Illustration of the Manhattan distance measurement used in AVC. Given that **x**_*k*_ is a misclassified instance in the intersection **S**. Then we can easily find out its nearest neighbors on both features **I**_*ik*_ and **I**_*jk*_. (**a**) shows that **I**_*ik*_ and **I**_*jk*_ are two separate instances. The *red solid line* represents the Manhattan distance between two pair of points (**x**_*k*_,**I**_*ik*_) and (**x**_*k*_,**I**_*jk*_). (**b**) shows that **I**_*ik*_ and **I**_*jk*_ are the same instance, so we discard **x**_*k*_ in the next calculation

To get the intersection **S**, we focus on the set of misclassified instances of each feature. All instances are ranked according to their values of feature **f**_*i*_ and get the rank of instances $\{\mathbf {x}_{r_{1}},\mathbf {x}_{r_{2}},\ldots,\mathbf {x}_{r_{n}}\}$. Then we consider the percentage of instances from each class in the sequence $\{\mathbf {x}_{r_{|n/2|}},\ldots,\mathbf {x}_{r_{n}}\}$ and define the class with larger percentage as the positive class. Clearly, we can simply classify the instances $\{\mathbf {x}_{r_{1}},\mathbf {x}_{r_{2}},\ldots,\mathbf {x}_{r_{n_{1}}}\}$ into the negative class and other instances into positive class. Then, we can easily distinguish the misclassified instances whose predictive information is inconsistent with the original one. For each instance **x**_*k*_ in **S**, we find the nearest miss **I**_*ik*_ from dimension **f**_*i*_ and **I**_*jk*_ from dimension **f**_*j*_. In the two-dimensional feature space, as shown in Fig. 3, we calculate the Manhattan distance between two pairs of points (**x**_*k*_,**I**_*ik*_) and (**x**_*k*_,**I**_*jk*_), and use the larger one to compute the complementarity. If **I**_*ik*_ and **I**_*jk*_ are different instances as shown in Fig. 3 (a), *dis*(**x**_*k*_,**I**_*ik*_) is taken as the complementarity, which is denoted as the red solid line in the figure. If **I**_*ik*_ and **I**_*jk*_ are the same instance as shown in Fig. 3 (b), the distance is not involved in complementarity. This implies that the two features provide little complementarity to each other in classifying instance **x**_*k*_.

In Eq. (4), the numerator of *h*_*ij*_ is the sum of distances over the instances in the intersection **S**, whose nearest misses are different according to the two features. The denominator of *h*_*ij*_ is the size of **S**. For any pair of strongly complementary features, the number of nonzero items in the numerator is equal or a little less than the size of **S**. But for the pair of features with weak complementarity, this number may much less than the size of **S**. Evidently, it is reasonable that *h*_*ij*_ can be used to measure the complementarity between two features.

**Fig. 4:**
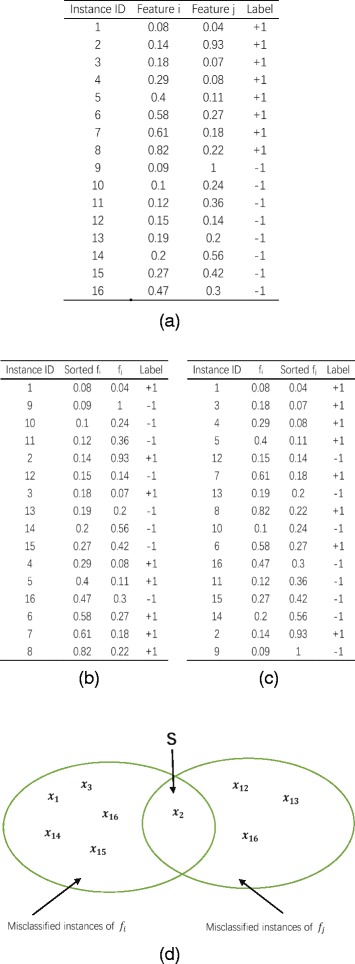
An example of artificial data set. (**a**) Shows the instances’ values in the data set. In (**b**), instances are sorted according to the value of feature **f**_*i*_. And in (**c**), instances are sorted according to the value of feature **f**_*j*_. (**d**) Shows the misclassified instances subset of sample data in (**a**), and **S** is the intersection of two subsets

We illustrate the computation process of the complementarity by using a simple example data set in Fig. 4 (a). The data set contains 16 instances, in which 8 instances belong to class “+1” and 8 instances to class “-1”. Figure 4 (b) and (c) show the ranking results of these instances. In Fig. 4 (b), the class “+1” is deemed as the positive class. Correspondingly, the class “-1” is deemed as the negative class. We classify the top-8 instances to class “+1”, and classify the other 8 instances to class “-1”. Then, we get the misclassified instances subset of **f**_*i*_ as {**x**_1_,**x**_2_,**x**_3_,**x**_14_,**x**_15_,**x**_16_}. In Fig. 4 (c), the class “-1” is taken as the positive one. So, the misclassified instances subset is obtained as {**x**_2_,**x**_6_,**x**_12_,**x**_13_}. The intersection **S** includes the only one instance **x**_2_, as shown in Fig. 4 d. **x**_2_ is an instance of class “+1”. In Fig. 4 (b), according to feature **f**_*i*_ we can find the nearest neighbor of **x**_2_ from class “-1” is instance **x**_12_. In Fig. 4 (c), according to feature **f**_*j*_, the nearest neighbor of **x**_2_ from the class “-1” is instance **x**_9_. The Manhattan distance between pairwise instances (**x**_2_,**x**_12_) is “0.8”, and the distance of (**x**_2_,**x**_9_) is “0.12”. It is obvious that the Manhattan distance between **x**_2_ and **x**_12_ is larger than that between **x**_2_ and **x**_9_. So we use the distance *dis*(**x**_2_,**x**_12_) to compute *h*_*ij*_ for feature **f**_*i*_ and **f**_*j*_.

The procedure of AVC is illustrated in Algorithm 1. Directly, we employ an efficient heuristic search strategy to select optimal features with highest complementarities. We select the most significant feature with the maximal AUC at the initial state. Then we iteratively select the features which have the maximal complementarities with the features selected in the prior state. In line 16 in Algorithm 1, when searching the optimal feature in the current state, we use the sum value of two features’ AUC as their complementarity weight. The purpose is that, for a certain feature, if there are more than one feature have the same complementarity with it, we prefer to the one with the maximal AUC value.

For the input data set containing *n* instances, the time complexity of calculating *m* features’ AUCs of line 2 in Algorithm 1 is *O*(*mnlogn*). For lines 3 to 6, selecting the top- *t* ^∗^ features costs *O*(*t* ^∗^ *logm*) time. Then, for lines 7 to 10, calculating *h*_*ij*_ for the *t* ^∗^ features costs *O*((*t* ^∗^)^2^) time. To get the optimal feature set, it takes *O*(*tt* ^∗^ *logt* ^∗^) for lines 14 to 20. Usually, the number of the candidate features *t* ^∗^ and the number of the selected features *t* is much smaller than *m* and *n*. Therefore, the complexity of the method is approximately equal to *O*(*mnlogn*+*t* ^∗^ *logm*).



### Multi-class problem

Our approach AVC can deal with not only the binary-class problem but also the multi-class problem. In this section, we use new strategies on the relevance analysis and complementarity analysis for the multi-class problem, which are different from those adopted in the binary-class problem.

As to the relevance analysis, we use MAUC to measure the relevance between features and target class. As a metric to measure the performance of classifiers, MAUC in Eq. (2) is the average AUC over all sub-problems that consist of pairwise classes. So in AVC, a multi-class problem is also divided into a batch of binary-class sub-problems in one-versus-one manner, in which each sub-problem consists of a pair of classes. A multi-class problem with *q* classes can be divided into $\frac {q(q-1)}{2}$ binary sub-problems. We use the same way as the binary-class problem to calculate the MAUC of features with Eq. (2).

In the complementarity analysis, we should get the misclassified instances by each feature. For each feature, it corresponds to a misclassified instance set for each binary-class sub-problem. We use **B** **S**_*ab*_(**f**_*i*_) to represent the misclassified instance set of feature **f**_*i*_ in a binary-class sub-problem with respect to the *ath* class and the *bth* class. And we define the union of a feature’s misclassified instances sets in all binary-class sub-problems as the global misclassified instances set, which is represent by Eq. (5):

5 $$\begin{array}{@{}rcl@{}} \mathbf{MS}(f_{i})= BS_{12}(f_{i}) \bigcup BS_{13}(f_{i}) \cdots \bigcup BS_{(q-1)q}(f_{i}) \end{array} $$

For each pair of features **f**_*i*_ and **f**_*j*_, the intersection **S** is defined as $\mathbf {S}= \mathbf {MS}(f_{i})\bigcap \mathbf {MS}(f_{j})$. Same as the binary-class problem, for each instance **x**_*k*_ in **S**, we find the nearest miss **I**_*ik*_ from feature **f**_*i*_ and **I**_*jk*_ from feature **f**_*j*_. Note that we only use the nearest one no matter which class it belongs to. If we use the nearest neighbors from every other classes, such as the ReliefF method, it may bring some useless information to the complementarity analysis. Suppose that some nearest misses of **x**_*k*_ have large distances **f**_*i*_, they may make little contributions to the analysis of the complementarity. In order to find the features with the optimal complementarity, we only pay attention to the nearest neighbor from the closest different class.

For the input multi-class data set with *n* instances characterized by *m* features and classified to *q* classes, the time complexity of calculating *m* features’ MAUC is *O*(*q* ^2^ *mnlogn*), corresponding to line 2 in Algorithm 1. Since the other steps have the same computational complexity as the binary-class problem, the complexity of our method for multi-class problem is *O*(*q* ^2^ *mnlogn*+*t* ^∗^ *logm*).

## Results and discussion

### Benchmark data sets

We use 13 publicly available microarray data sets to evaluate the performance of the selected features, as shown in Table 1. These data sets are widely used in the studies of gene selection problems [31–33].

**Table 1:** Benchmark data sets

Data set	*♯* Features	*♯* Instances	*♯* Classes
Colon (COL)	2000	62	2
Lymphoma (LYM)	4026	96	9
ALL-AML-4 (ALL)	7129	72	4
CNS	7129	60	2
Leukemia (LEK)	7192	72	2
Carcinom (CAR)	9182	174	11
Breast-5 (BR5)	9217	84	5
CLL-SUB-111 (CLL)	11340	111	3
MLL	12582	72	3
Lung Cancer (LUN)	12600	203	5
Ovarian (OVA)	15154	253	2
GLI-85 (GLI)	22283	85	2
Breast Cancer (BRC)	22481	97	2

### Comparisons with the state-of-the-art methods

#### FAST

FAST [16] is a feature selection method for small samples and imbalanced data classification problems. It directly calculates the AUC of each feature by plotting the ROC curve and summing up the area under it. For small samples data, in order to avoid the redundant thresholds, FAST divides instances into *K* bins according to instances’ values and fixes the number of instances to fall in each bin. Then, the mean of instances in each bin is used as the threshold to get the point (FPR, TPR) on the ROC curve. After ranking the features according to their AUCs in descending order, the top-k features are selected. Although FAST can perform well for some microarray data sets on SVM and 1-NN classifiers, the computation process of AUC is complex and imprecise. Besides, FAST does not take into account the redundancy in the feature set. FAST can find the most significant features to discriminate given two classes, however, the selected features are sometimes too redundant. And previous studies have emphasized that considering both relevance and redundancy in the feature selection procedure leads to better feature subset in most cases [19].

#### ARCO

For overcoming the problems in the FAST feature selection method, Wang et al. [19] proposed ARCO feature selection method. ARCO uses Eq. (1) to calculate the AUC for each feature. In this way, ARCO not only guarantees the precision of the AUC, but also simplifies the computational process. Moreover, ARCO removes the redundant features using the Spearman’s Rank Correlation Coefficient (RCC). Given two features **f**_1_ and **f**_2_, ARCO sorts the instances on each feature based on their values. RCC can be calculated by Eq. (6):

6 $$\begin{array}{@{}rcl@{}} RCC(\mathbf{f}_{1},\mathbf{f}_{2})=1-\frac{6\sum\limits_{i=1}^{n}d_{i}^{2}}{n\times(n^{2}-1)}, \end{array} $$

where *d*_*i*_ is the difference between an instance **x**_*i*_’s ranks on two features, and *n* is the number of instances.

To select *k* features from the whole feature set whose size is *m*, ARCO starts from the feature with the largest AUC. It iteratively evaluates every previously unselected feature **f**_*i*_ with Eq. (7), and selects the feature with the largest value of *E*(**f**_*i*_):

7 $$\begin{array}{@{}rcl@{}} E(\mathbf{f}_{i})=AUC(\mathbf{f}_{i})-\frac{\left|\sum\limits_{\mathbf{f}_{j}\in\mathbf{S}}RCC(\mathbf{f}_{i},\mathbf{f}_{j})\right|} {|\mathbf{S}|}, \end{array} $$

where *AUC*(**f**_*i*_) is the AUC when taking the single feature **f**_*i*_ as a classifier, **S** is the current selected feature subset, and |**S**| is its cardinality.

In every iteration, AROC selects the feature with the smallest redundancy to the features in the subset. The redundancy is represented by the RCC, which mainly shows the different positions of instances on the two features’ ranking sequences. For two features, the large the difference is, the small the redundancy is. Consider an extreme situation, two features can both classify all instances from two classes. On one feature, the values of instances from one class are all larger than instances from the other class, but on the other feature these values are smaller than the others. We can see that ranks of the instances are totally different on the two features, so the RCC of them indicates that they are not redundant. But to build a classifier, any one of them is enough to separate all instances. So sometimes ARCO cannot exactly recognize the redundant features. And it is necessary to differentiate the correctly classified and misclassified instances by each feature.

#### FROC

Another feature selection method based on ROC analysis is FROC [21], which is developed to overcome the redundancy problem in small samples microarray data sets. This method also has two steps. The first step is a one-gene-at-a-time filtering which uses the ROC curve as a criterion to evaluate the relevance of features to the target class. Different from ARCO, FROC chooses to calculate the area between the ROC curve and the diagonal line (ARD), which is equal to *AUC*−0.5. Instances are also sorted in increasing order according to the values for feature *f*_*i*_ and *ARD*(*f*_*i*_) is calculated by Eq. (8):

8 $$\begin{array}{@{}rcl@{}} {}\mathbf{ARD}(f_{i})\,=\, \frac{\left|\sum_{i=1}^{n_{1}}\!\left(q_{i}\,-\,2i\right)\!\right|}{n_{0}\!\times\! n_{1}} \,=\, \frac{\left|\left(\sum_{i=1}^{n_{1}}{q}_{i}\right)\,-\,n_{1}\!\times\!(n_{1}\,+\,1)\!\right|}{n_{0}\!\times\! n_{1}} \end{array} $$

where *n*_0_ and *n*_1_ are the numbers of positive and negative instances respectively, and *q*_*i*_ is the rank of the *i*th negative instance. All features are sorted by the *ARD*(*f*_*i*_) of feature *f*_*i*_ in descending order and the top of the sorted features are chosen as a candidate feature set. The second step in FROC is a ROC-curve-based Markov blanket filtering. This step removes the redundant features using the definition of Markov blanket that if **M**_*i*_ is a Markov blanket of **f**_*i*_, the probabilistic distribution *P* of classes is invariant under no matter what value **f**_*i*_ takes:

$$P\left(\mathbf{F}\,-\,\mathbf{M}_{i}\,-\,\{\mathbf{f}_{i}\},\mathbf{C}|\mathbf{f}_{i},\mathbf{M}_{i}\right)\,=\,P\left(\mathbf{F}\,-\,\mathbf{M}_{i}\,-\, \{\mathbf{f}_{i}\},\mathbf{C}|\mathbf{M}_{i}\right). $$

**Fig. 5:**
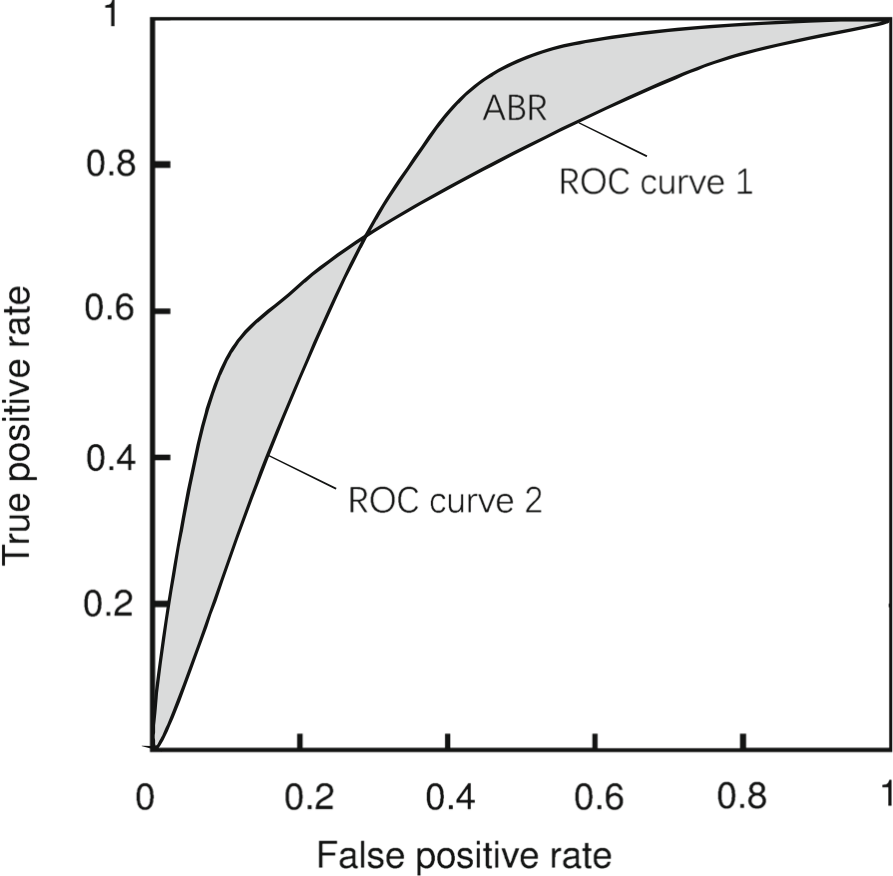
ABR of two features. The *gray* area is the ABR area, and *two curves* are the ROC curves respectively obtained by two features

FROC uses the area between the ROC curves (ABR) to measure the redundancy of two features. For example, ABR of two features is the gray area in Fig. 5. The smaller the ABR is, the more redundant the two features are. FROC iteratively removes the redundant features from the candidate feature set selected in the first step.

In [21], the author argued that it is not able to find an exact Markov blanket of a given feature. The alternative method is to find an approximation to Markov blanket of the feature. This may cause a problem that after finding out the redundant features, removing different features may bring different influence to the combination of features in subset when building the classifier. To overcome this problem, the analysis on the complementarity of feature classification capability maybe a feasible choice.

### Experimental settings

The efficacy of our new method AVC was empirically evaluated by comparing it to four state-of-the-art feature selection methods. Three methods, FAST, ARCO and FROC, are all based on the ROC curve and AUC. These three methods are all particularly designed for the binary-class classification problems. So in our experiments, we extend them to solve the multi-class classification problems with the same strategy as our method. That is, for the multi-class problem, the MAUC of features will be computed by Eq. (2). The fourth method is ReliefF, which has been widely used as the compared algorithm that uses the criterion of preserving sample similarity [34]. We compare the performance on four widely used classifiers to test the robustness of the five methods. The classifiers are Naive Bayes, Support Vector Machine (SVM), 1-Nearest Neighbor (1-NN) and C4.5 Decision Tree. Due to the small number of instances in these microarray data sets, we use 10-fold cross-validation to evaluate the classification performance of the classifiers.

We perform our comparisons in two sub-experiments. In the first sub-experiment, we compare four feature weighting methods, i.e., AVC, FAST, ARCO and ReliefF. These methods select features according to their weights, so we evaluate their classification performance in the condition of increasing the number of features. In the second sub-experiment, we evaluate their classification performance in the condition of fixing the number of features determined by FROC. FROC is a method which selects a feature subset rather than evaluating features individually, so we fix the number of features to the size of the feature subset selected by FROC.

To avoid the influence of the imbalanced class issue on the classification accuracy, we choose the balance error rate (BER) metric [16] to evaluate the performance of the classifiers on both classes for the binary-class problem, which is defined as follows:

9 $$\begin{array}{@{}rcl@{}} BER=\frac{1}{2}\left(\frac{FP}{FP+TP}+\frac{FN}{FN+TN}\right), \end{array} $$

where *FP*, *TP*, *FN*, and *TN* are respectively the false positive, the true positive, the false negative, and the true negative. If the classes are balanced, BER is equal to the global error rate. For the multi-class problem, BER can be computed as follows:

10 $$\begin{array}{@{}rcl@{}} BER'=\frac{1}{q}\sum\limits_{l=1}^{q}\frac{n_{f_{l}}}{n_{l}}, \end{array} $$

where *n*_*l*_ is the number of the instances in the class **c**_*l*_, and $n_{f_{l}}$ is the number of the misclassified instances in **c**_*l*_. Another evaluation statistic commonly used on microarray data sets is the area under the ROC (AUC). This statistic is similar in nature to the BER in that it weights errors differently on the classes. Then, we explore the Wilcoxon signed-rank test to compare AVC with the other three methods, and the significance level is set to 0.05.

We used the well-known WEKA software package [35] as our experiments’ platform. Our method and other compared methods are all implemented at this platform. For FAST and ReliefF, we select the top-100 features as the final feature subset. For ARCO and our method, we select the top-200 features as the candidate feature subset, and select the top-100 features as the final feature subset. For FROC, we also select the top-200 features as the candidate feature subset and the final feature subset is selected from these features. In ReliefF, every instance is used to update the weights of features and for every instance we find ten nearest neighbors from both the same class and the different classes.

### Experimental analysis

**Fig. 6:**
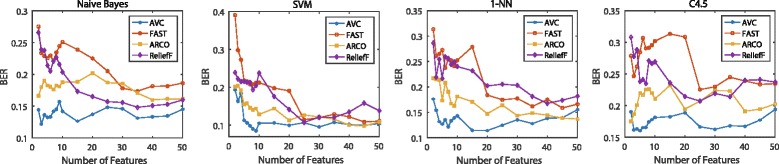
Averaged BER on binary-class data sets. Averaged BER value of the four classifiers on the six binary-class data sets using four classifiers. We choose 17 feature subsets with increasing number of features

**Fig. 7:**
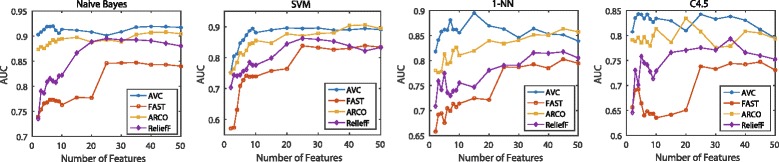
Averaged AUC on binary-class data sets. Averaged AUC value of the four classifiers on six binary-class data sets using four classifiers. We choose 17 feature subsets with increasing number of features

**Fig. 8:**
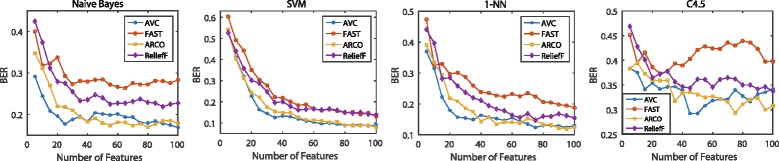
Averaged BER on multi-class data sets. Averaged BER value of the four classifiers on seven multi-class data sets using four classifiers. We choose 20 feature subsets with increasing number of features

**Fig. 9:**
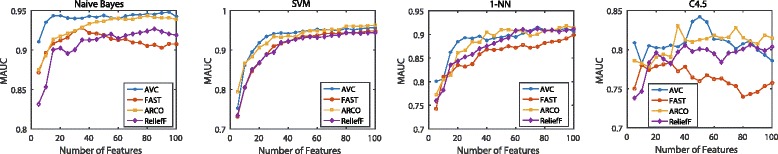
Averaged MAUC on multi-class data sets. Averaged BER value of the four classifiers on seven multi-class data sets using four classifiers. We choose 20 feature subsets with increasing number of features

The classification performance is illustrated in Figs. 6, 7, 8 and 9. For the binary-class classification problems, we test across the six binary-class data sets shown in Table 1, which are COL, CNS, LEK, OVA, GLI and BRC. We examine 17 groups of features with different size in each test. When the size is smaller than 10, we add a feature every time. After the size is larger than 10, we add five features every time until the size is equal to 50. Then the averaged performance of each classifier with each data set is calculated. Figures 6 shows the BER scores for the six binary-class data sets with respect to the four classifiers. We also use AUC to evaluate the classifiers on test data. Figure 7 shows the AUC scores averaged over the six binary-class data sets with four chosen classifiers. For the multi-class classification problems, we experiment on the seven multi-class data sets in Table 1, i.e., LYM, ALL, CAR, BR5, CLL, MLL and LUN. We examine 20 groups of features with different size in each test, and every time we add 5 features. Same as the binary-class classification problems, we also use the BER and AUC to measure the performance of classifiers. Figure 8 shows the BER scores for the seven multi-class data sets with four classifiers and Fig. 9 shows the MAUC scores averaged over these seven multi-class data sets.

The average results in Figs. 6 and 7 for binary class classification problem demonstrate that AVC significantly outperforms the other compared methods. The features selected by AVC reach the best performance with less than 15 features, which are much smaller than the number of the features selected by other three feature selection methods. And with more than 15 features, although AVC features do not improve the BER metric or AUC metric of the classifiers, its performance is still better than the three compared feature selection methods. Our method is based on the analysis of the ROC and AUC, so it is reasonable to believe that a learning method using AVC-selected features would also maximize the AUC.

The average results in Figs. 8 and 9 for multi-class classification problems show that AVC features also performe well when the size of feature subset is small. When using the Naive Bayes classifier, SVM classifier and 1-Nearest Neighbor classifiers, with less than 35 features AVC performs better than the other three feature selection methods. With more than 35 features, the differences between AVC and the other compared algorithms are not significant. When using the C4.5 Decision Tree classifier, feature subsets selected by different methods perform much different. The feature subsets selected by AVC get the best performance when their size is about 50, which is better than other three methods for all 20 different sizes of feature subsets.

Table 2 shows the minimal BER of the four classifiers with top-100 features on the benchmark data sets. In Table 2, we can see that AVC can get the minimal BER in a majority of the situations. Table 3 shows the size of feature subsets selected by four methods when four classifiers get the minimal BER with top-100 features. We can see that, AVC is capable of choosing a smaller size of feature subset than other three feature selection methods for the binary-class problem. But for the multi-class problem, it is hard to say AVC can always choose the minimal size of the features. These may mainly because of the influence of the well-known “siren pitfall” in scoring methods for multi-class problem, which is common to feature-scoring methods which focus on selecting the top scoring features [36].

**Table 2:** Minimal BER of the four classifiers in the top-100 features on the benchmark data sets

Classifier	Algorithm	Data sets												
		*COL*	*LYM*	*ALL*	*CNS*	*LEK*	*CAR*	*BR5*	*CLL*	*MLL*	*LIM*	*OVA*	*GLI*	*BRC*
NB	*AVC*	**0.125**	**0.121**	**0.097**	0.2	**0**	0.207	0.073	**0.186**	**0.012**	**0.07**	**0.009**	**0.046**	**0.135**
	*FAST*	0.172	0.395	0.332	0.194	0	0.331	0.144	0.188	0.069	0.161	0.036	0.142	0.187
	*ARCO*	0.135	0.24	0.159	**0.13**	0	**0.199**	**0.014**	0.189	0.036	0.079	0.031	0.168	0.193
	*ReliefF*	0.135	0.247	0.159	0.2	0.141	0.212	0.16	0.245	0.012	0.125	0.009	0.089	0.165
SVM	*AVC*	**0.085**	**0**	**0.03**	0.101	**0**	**0.051**	0.006	0.134	**0.012**	0.085	**0**	0.054	0.116
	*FAST*	0.106	0.186	0.132	0.125	0	0.173	0.052	**0.079**	0.029	.056	0.003	0.104	**0.078**
	*ARCO*	0.111	0	0.115	**0.044**	0.01	0.055	**0**	0.12	0.042	**0.054**	0	**0.046**	x0.196
	*ReliefF*	0.111	0.086	0.069	0.169	0.057	0.08	0.143	0.194	0.012	0.082	0	0.065	0.132
1-NN	*AVC*	0.135	**0.005**	**0.036**	0.148	0.019	**0.089**	**0.019**	0.153	0.031	0.113	**0.003**	0.046	**0.135**
	*FAST*	0.141	0.198	0.201	0.289	**0**	0.247	0.081	**0.139**	0.052	0.121	0.003	0.089	0.214
	*ARCO*	**0.107**	0.018	0.127	0.149	0	0.098	0.019	0.181	0.056	**0.063**	0.003	0.073	0.173
	*ReliefF*	0.124	0.086	0.046	**0.142**	0.221	0.085	0.074	0.18	0.029	0.075	0.003	**0.045**	0.145
C4.5	*AVC*	**0.073**	**0.362**	0.167	**0.236**	0.104	0.289	0.136	**0.193**	0.107	0.259	0.019	**0.065**	0.193
	*FAST*	0.162	0.455	0.332	0.22	**0.052**	0.395	0.259	0.227	0.069	0.369	0.021	0.142	**0.016**
	*ARCO*	0.156	0.44	**0.138**	0.236	0.092	0.326	**0.121**	0.21	0.107	0.247	**0.014**	0.134	0.227
	*ReliefF*	0.12	0.395	0.268	0.333	0.152	**0.265**	0.308	0.237	**0.06**	**0.153**	0.016	0.13	0.227

Bold data in the table reflect the minimal BER of four classifiers in the top-100 features selected by four compared feature selection methods on the benchmark data sets

**Table 3:** Size of the selected feature subsets when BER is minimal with top-100 features

Classifier	Algorithm	Data sets												
		*COL*	*LYM*	*ALL*	*CNS*	*LEK*	*CAR*	*BR5*	*CLL*	*MLL*	*LIM*	*OVA*	*GLI*	*BRC*
NB	*AVC*	10	40	12	34	39	21	72	12	9	18	17	22	3
	*FAST*	12	25	6	6	4	33	63	58	28	32	78	12	7
	*ARCO*	78	39	25	42	3	84	68	4	47	98	4	69	83
	*ReliefF*	6	32	8	8	37	41	82	86	22	76	66	18	7
SVM	*AVC*	7	39	93	5	4	78	72	34	99	62	42	10	3
	*FAST*	52	83	89	84	4	99	79	96	91	98	98	39	79
	*ARCO*	35	100	30	42	5	92	52	86	76	74	56	72	19
	*ReliefF*	23	87	33	34	97	81	57	91	55	66	23	13	19
1-NN	*AVC*	64	85	29	14	4	80	72	8	27	52	11	18	16
	*FAST*	26	82	100	28	4	99	62	98	36	99	98	50	6
	*ARCO*	28	79	77	66	5	83	55	40	48	63	92	98	99
	*ReliefF*	95	80	15	41	70	99	97	64	60	77	26	72	31
C4.5	*AVC*	27	46	7	53	3	57	65	44	1	13	14	24	3
	*FAST*	9	20	26	47	3	69	23	26	4	18	89	28	4
	*ARCO*	15	34	72	18	3	76	93	23	1	75	17	55	17
	*ReliefF*	21	66	40	3	19	45	30	40	29	81	34	31	3

**Fig. 10:**
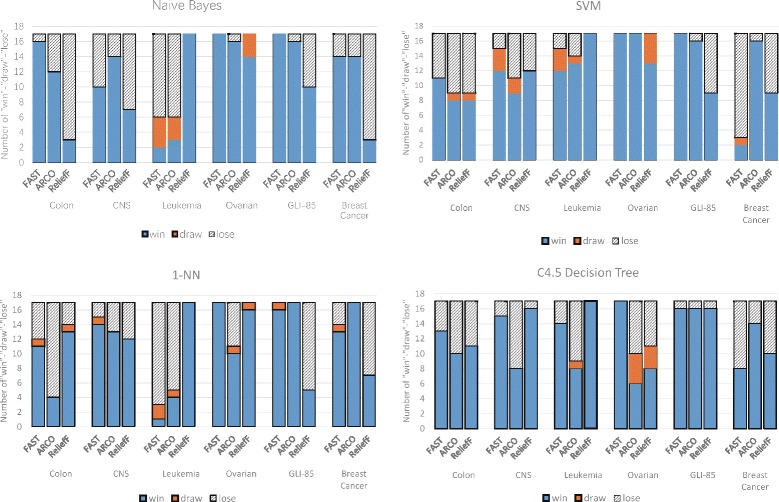
Wilcoxon signed-rank tests on binary-class data sets. The results of the Wilcoxon signed-rank tests on six binary-class data sets with 17 groups of selected features

**Fig. 11:**
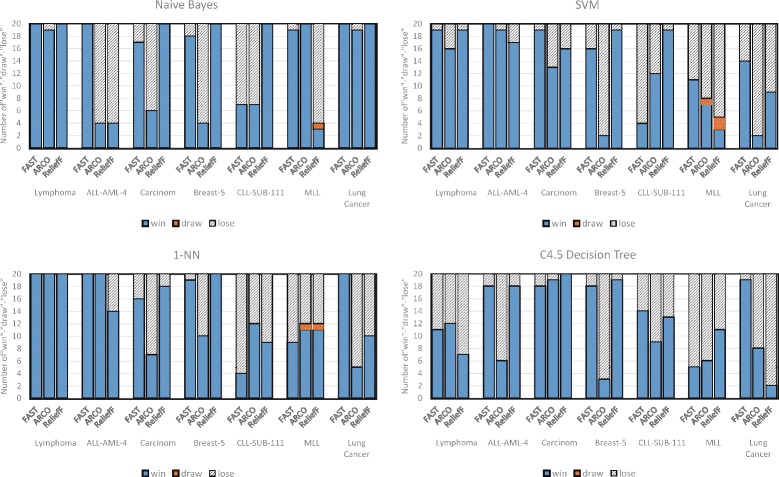
Wilcoxon signed-rank tests on multi-class data sets. The results of the Wilcoxon signed-rank tests on seven binary-class data sets with 20 groups of selected features

Figure 10 presents the results of the Wilcoxon signed-rank tests on 17 groups of the binary-class data sets, and Fig. 11 presents that for 20 groups of the multi-class data sets. In the figures, “win” indicates the number of the cases in which AVC is significantly better than the compared algorithms, “draw” indicates that AVC performs identically, and “lose” indicates that AVC performs worse. From the figures, we can observe that in a majority of the cases, AVC performs superior or comparable to the other methods.

**Fig. 12:**
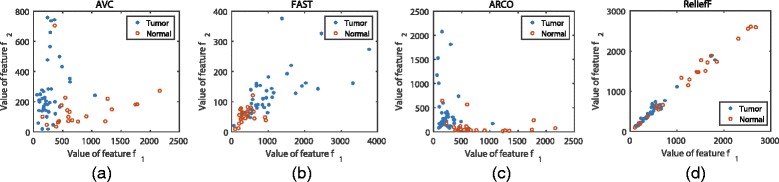
Instances distribution of Colon cancer data with the two best features selected from four feature selection methods, (**a**) shows the instances’ distribution on the best two features selected by AVC, (**b**) shows the instances’ distribution on the best two features selected by FAST, (**c**) shows the instances’ distribution on the best two features selected by ARCO, and (**d**) shows the instances’ distribution on the best two features selected by ReliefF

**Fig. 13:**
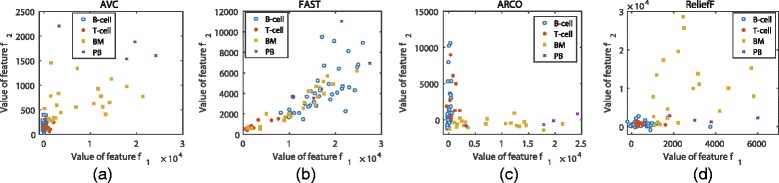
Instances distribution of ALL-AML-4 data with the two best features selected from four feature selection methods, (**a**) shows the instances’ distribution on the best two features selected by AVC, (**b**) shows the instances’ distribution on the best two features selected by FAST, (**c**) shows the instances’ distribution on the best two features selected by ARCO, and (**d**) shows the instances’ distribution on the best two features selected by ReliefF

Figures 12 and 13 show the class distributions of the Colon cancer data and ALL-AML-4 data with the two best features selected by four methods, respectively. The classes in Figs. 12 (a) and 13 (a) are scattered and have little overlapping, which makes it easy to find the optimal boundaries between them. But in Figs. 12 (b) to (d) and 13 (b) to (d), instances from different classes are overlapping so that it is difficult to classify them by some certain boundaries. This may explain why our method can perform well with a small size of feature subsets.

Table 4 shows the averaged BER and AUC of the four classifiers for the five feature selection methods. Note that the number of the selected features is determined by FROC, which can determine the number of the selected features. For example, FROC selected a feature subset from the Colon data set which includes 69 features. To compare the performance with other four methods, we fix the size of feature subset to 69. From Table 4 we can see that AVC is comparable or superior to the other compared methods.

**Table 4:** Averaged BER and AUC of the four classifiers on the benchmark data sets

		*AVC*	*FAST*	*ARCO*	*Relief*	*FROC*
BER	*NB*	**0.184**	0.242	0.194	0.209	0.269
	*SVM*	0.148	0.166	**0.147**	0.174	0.201
	*1-NN*	0.163	0.213	**0.162**	0.178	0.254
	*C4.5*	**0.28**	0.352	0.287	0.296	0.299
AUC	*NB*	**0.924**	0.894	0.911	0.894	0.881
	*SVM*	0.896	0.908	**0.913**	0.886	0.898
	*1-NN*	0.873	0.855	**0.874**	0.861	0.822
	*C4.5*	**0.798**	0.766	0.791	0.788	0.793

Bold data in the table reflect the minimal averaged BER and AUC of four classifiers in the feature subset selected by five compared feature selection methods on the benchmark data sets

### Evaluation with LDA and Mclust

Some classifiers can account for the high correlations among features appropriately, such as LDA (Linear Discriminant Analysis) and Mclust (Model-based Clustering method). In this sub-experiment, we further evaluate the performance of AVC on this kind of classifiers.

**Fig. 14:**
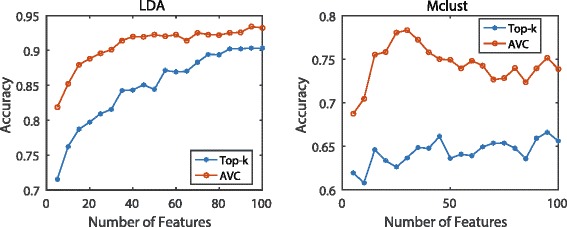
Averaged accuracy of the LDA and Mclust classifiers. Averaged accuracy value of the LDA and Mclust classifiers on thirteen data sets. We choose 20 feature subsets with increasing number of features

We experiment across the thirteen data sets shown in Table 1. We examine 20 groups of features with different sizes and increase the number of features from 5 to 100 in interval of 5. Figure 14 shows the averaged accuracy of the thirteen data sets. The blue line named as Top-k reflects the performance of the top-k features with maximal AUC. Features are sorted according to their AUC scores and the top-k features are selected without any redundancy reduction process. The red line reflects the performance of AVC. We can observe that AVC leads to higher accuracy in all the cases.

Besides, FAST is a feature selection approach which simply selects the top-k features with maximal AUCs. Generally speaking, as shown from Figs. 6, 7, 8, and 9, it is clear that FAST performs inferior to the other feature selection methods, which involve feature redundancy or complementarity analysis in their selection processes. Thus, we can draw the conclusion that reducing feature redundancy or improving feature complementarity conduces to better recognition performance. The feature selection methods exploiting these tricks outperform the top-k methods without any further evaluation strategies. This property still holds on the situations that the feature-correlation-based classifiers are employed for measuring the discriminative performance of the selected features.

## Conclusion

We propose a new feature selection method specific to the recognition problems in the microarray data sets. This method ranks the features according their relevance to the class label and the complementarity between each other. The ROC curve and the area under the ROC curve (AUC) are exploited to evaluate the relevance between a feature and the class label. Then the distribution of data on a pair of features is analyzed to measure the complementarity of the pair of features. Moreover, the greedy searching strategy is also implemented for finding out the predominant features.

The experiment results show that when the number of selected features is small, the features selected by our method can achieve a better classification performance compared with the state-of-the-art methods. Moreover, it is illustrated from the experiments that the reduced subspace constructed by our new method is suitable for the recognition task, in which the classes are mostly separated from each other and a significant boundary between classes can be easily found.

## Abbreviations

ABR: Area between the ROC curves
ARD: Area between the ROC curve and the diagonal line
ARCO: AUC and rank correlation coefficient optimization
AUC: Area under the ROC curve
AVC: AUC-based variable complementarity
BER: Balance error rate
FAST: Feature assessment by sliding thresholds
FPR: False positive rate
FROC: Feature selection based-on ROC-curves
KNN: k-Nearest neighbor
RCC: Spearman’s rank correlation coefficient
ROC: Receiver operator characteristic
LDA: Linear discriminant analysis
MAUC: multi-class AUC
Mclust: Model-based clustering method
SVM: Support vector machine
TPR: True positive rate
